# Supplementary material for: AmpliconReconstructor integrates NGS and optical mapping to resolve the complex structures of focal amplifications
Source: Nat Commun. 2020 Sep 1;11:4374. doi: 10.1038/s41467-020-18099-z (PMC7463033; doi:10.1038/s41467-020-18099-z)
Supplement: Supplementary file 1 — Supplementary Information [file 41467_2020_18099_MOESM1_ESM.pdf]

Supplementary Information

AmpliconReconstructor integrates NGS and optical mapping to resolve the complex structures of focal amplifications

Luebeck et al.

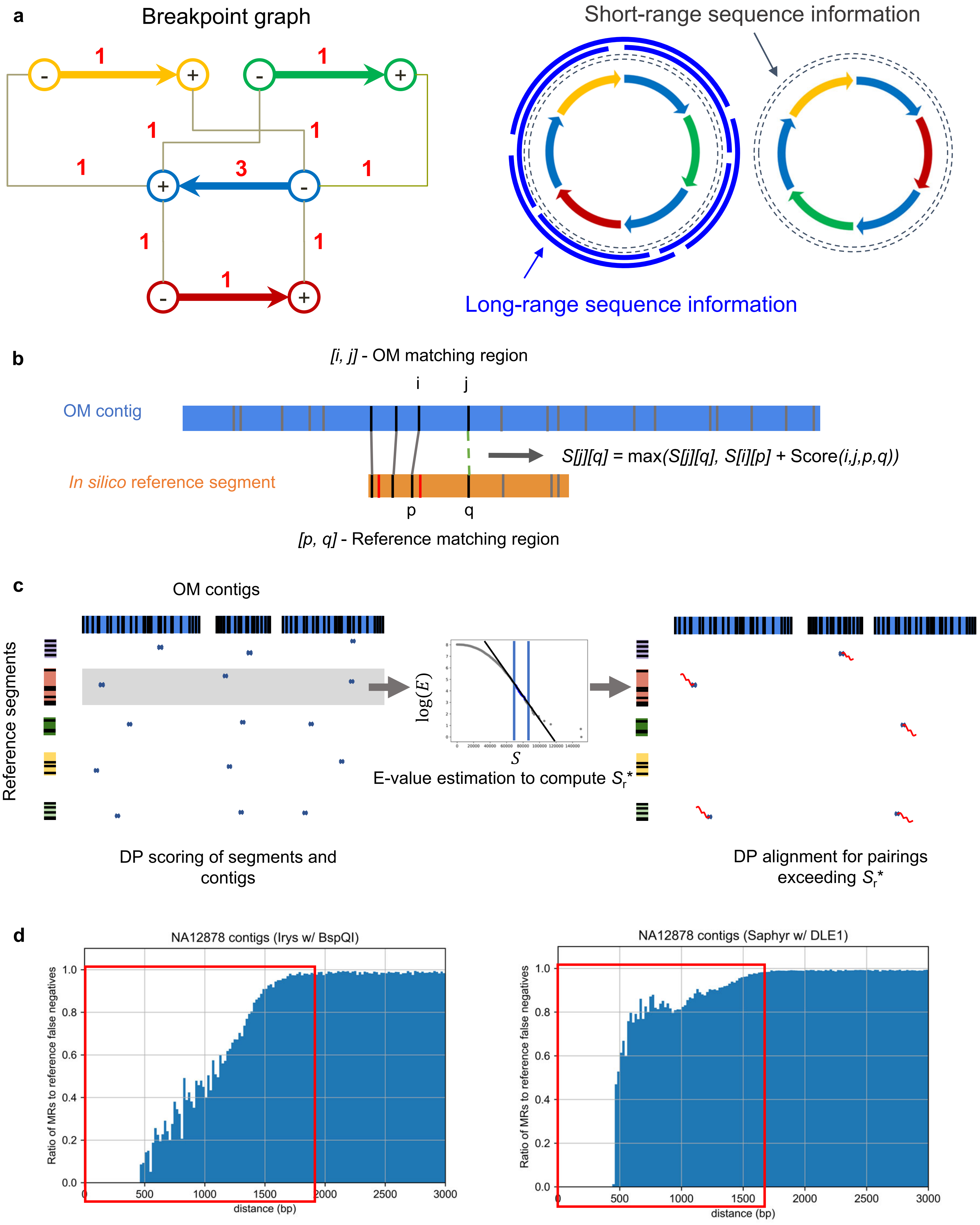
a
Breakpoint graph
1
1
1
1
1
3
1
1
1
1
Short-range sequence information
Long-range sequence information
b
[i, j] - OM matching region
i
j
OM contig
S[j][q] = max(S[j][q], S[i][p] + Score(i,j,p,q))
In silico reference segment
p
q
[p, q] - Reference matching region
c
OM contigs
Reference segments
log(E)
S
E-value estimation to compute S_r*
DP scoring of segments and contigs
DP alignment for pairings exceeding S_r*
d
NA12878 contigs (Irys w/ BspQI)
Ratio of MRs to reference false negatives
distance (bp)
NA12878 contigs (Saphyr w/ DLE1)
Ratio of MRs to reference false negatives
distance (bp)

Supplementary Figure 1. Motivation for AR and the SegAligner methods.

a, Long-range sequencing enables accurate disambiguation of large duplications. A breakpoint graph is represented on the left, with colored arrows representing oriented genomic segments, and with edges connecting the segments between source (+) and destination (-) breakpoints. The reconstructions on the right demonstrate that a graph containing duplicated segments can produce multiple possible reconstructions. Short reads provide information about individual junctions in the graph, but long-range sequence information can span and disambiguate multiple junctions. **b**, Diagram of optical map alignment between an OM contig and a reference segment. Unmatched labels prior to the current iteration's best preceding pairings are indicated in red. The SegAligner dynamic programming (DP) recurrence is shown for the matching regions *[i, j]* and *[p, q]*. **c**, The SegAligner method for computing statistically significant scoring alignments by estimating parameters in an E-value model from alignments of segments against all contigs present in a sample. An all vs. all representation of best alignment scores of genomic reference segments and OM contigs is shown on the left. The collection of scores of each reference segment are used to build a scoring distribution for the E-value model. In the diagram on the right, alignments are generated from the segment-contig pairings with significantly high-scoring alignment scores. **d**, Ratio of matching regions (MRs) to false-negative reference labels in Bionano NA12878 data indicates the distance-dependent rate of label collapse for consecutive labels in the hg19 reference genome. Two distinct patterns of label collapse exist between Bionano Irys (left) and Bionano Saphyr (right) platforms. Red boxes indicate the regions in the respective technologies that are considered by SegAligner to have non-zero probability of label collapse. Source data are provided as a Source Data file.

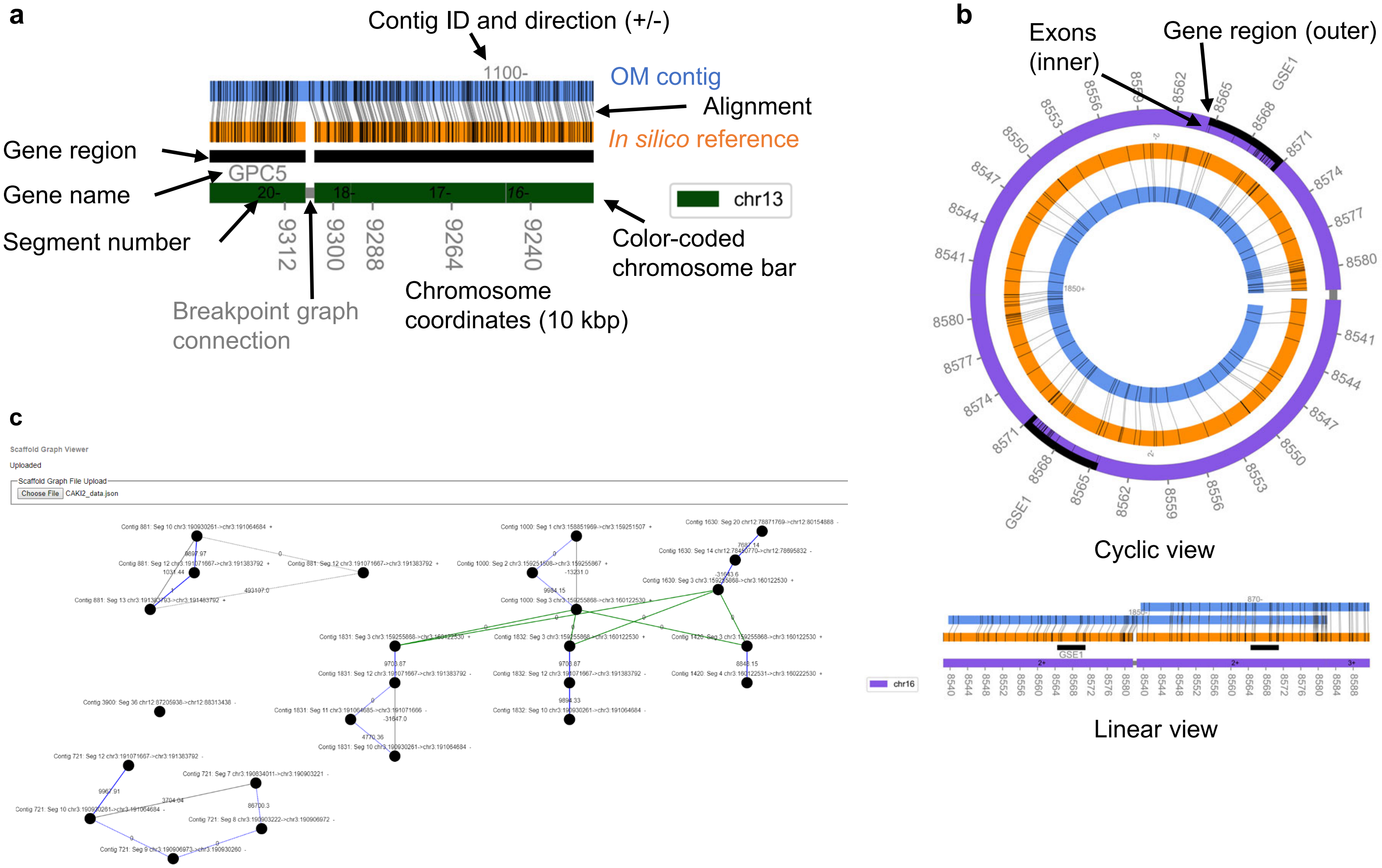

Supplementary Figure 2. Visualization of AR results.

a, Diagram of features in CycleViz visualization and associated feature tracks. **b**, CycleViz can generate both cyclic (top) and linear (bottom) visualizations. **c**, ScaffoldGraphViewer visualization of AR scaffold graphs using CytoscapeJS. Grey edges indicate connections in the scaffold directed acyclic graph (DAG). Dotted edges indicate imputation. Blue edges indicate edges used in the scaffold's heaviest path. Green edges indicate a link between two scaffolds.

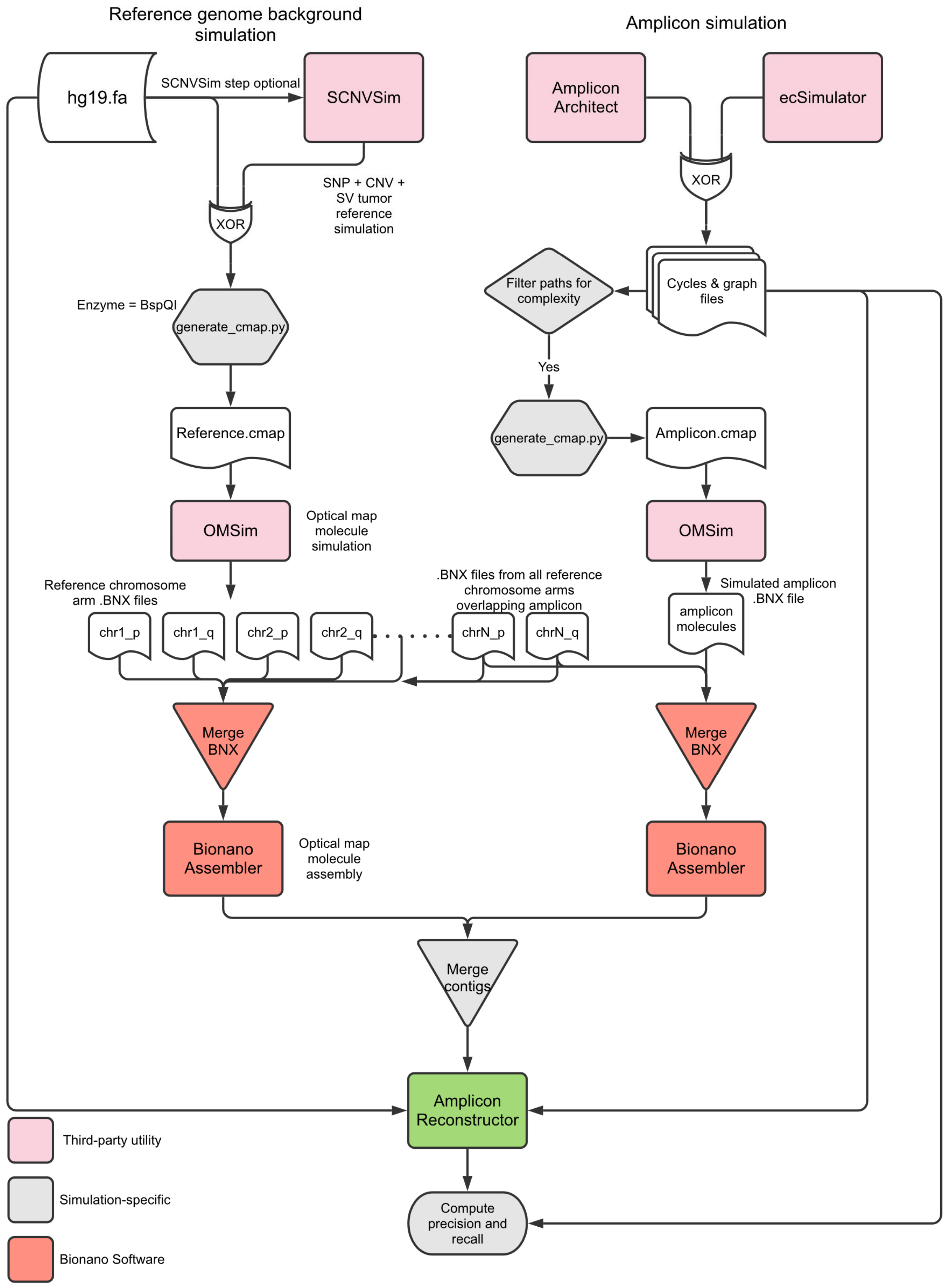

Reference genome background simulation
Amplicon simulation
hg19.fa
SCNVSim step optional
SCNVSim
Amplicon Architect
ecSimulator
XOR
SNP + CNV + SV tumor reference simulation
XOR
Cycles & graph files
Filter paths for complexity
Yes
Enzyme = BspQI
generate_cmap.py
Reference.cmap
generate_cmap.py
Amplicon.cmap
OMSim
Optical map molecule simulation
OMSim
Reference chromosome arm .BNX files
.BNX files from all reference chromosome arms overlapping amplicon
Simulated amplicon .BNX file
chr1_p
chr1_q
chr2_p
chr2_q
chrN_p
chrN_q
amplicon molecules
Merge BNX
Merge BNX
Bionano Assembler
Optical map molecule assembly
Bionano Assembler
Merge contigs
Amplicon Reconstructor
Compute precision and recall
Third-party utility
Simulation-specific
Bionano Software

Supplementary Figure 3. AR simulation study process diagram.

Process diagram and control flow for the AR simulation study. The simulations used two primary choices of reference genome, hg19 or hg19 simulated tumor (using SCNVSim). Two choices of amplicon sources are provided; those generated by AA on cancer cell lines (85 cases) or circular ecDNA simulated by ecSimulator (20 cases). Molecules are simulated from background reference and amplicon separately using OMSim. The resulting BNX files are merged such that the amplicon molecules also get the simulated reference molecules from all overlapping chromosome arms. The merged BNX files for amplicon and background reference are assembled independently. This way the background reference assembly only needs to be generated once for all amplicons used. The contigs from the background reference and amplicon are then merged. AR is run on the merged contigs, the breakpoint graph that was either simulated or selected from AA as well as the *in silico* reference genome. Pink filling indicates “third-party” utilities, which are not part of AR or the simulation pipeline. Grey filling denotes tools and scripts developed specifically for the simulation process. Salmon-colored filling indicates tools released by Bionano Genomics, Inc. (San Diego, CA).

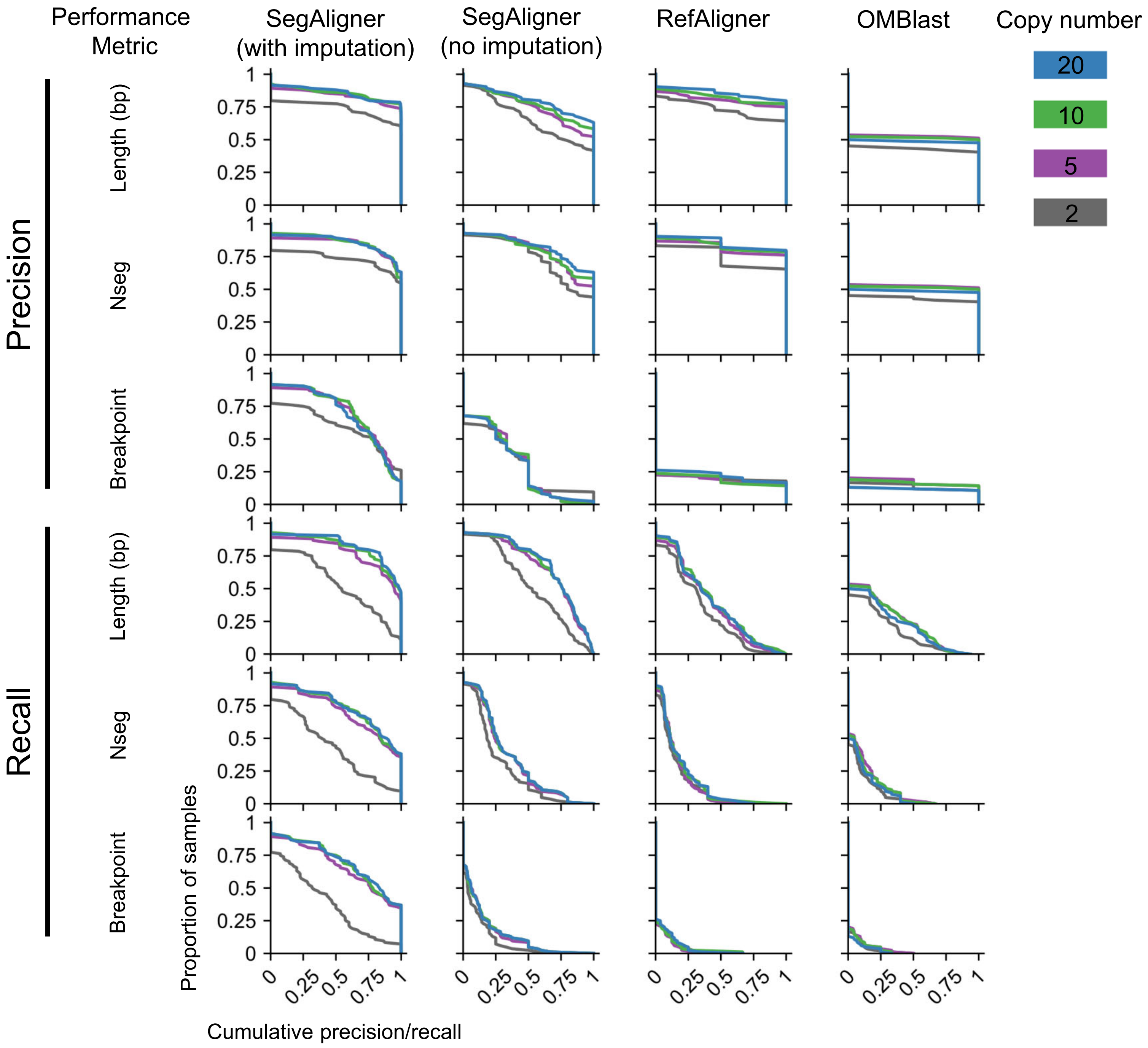

Supplementary Figure 4. AR performance on simulated data using different OM alignment techniques.

Cumulative precision and recall curves measuring performance of AR reconstructions on simulated amplicon OM data using different OM alignment methods and precision/recall measurement methods. OM assembly failures were not filtered from this figure. Source data are provided as a Source Data file.

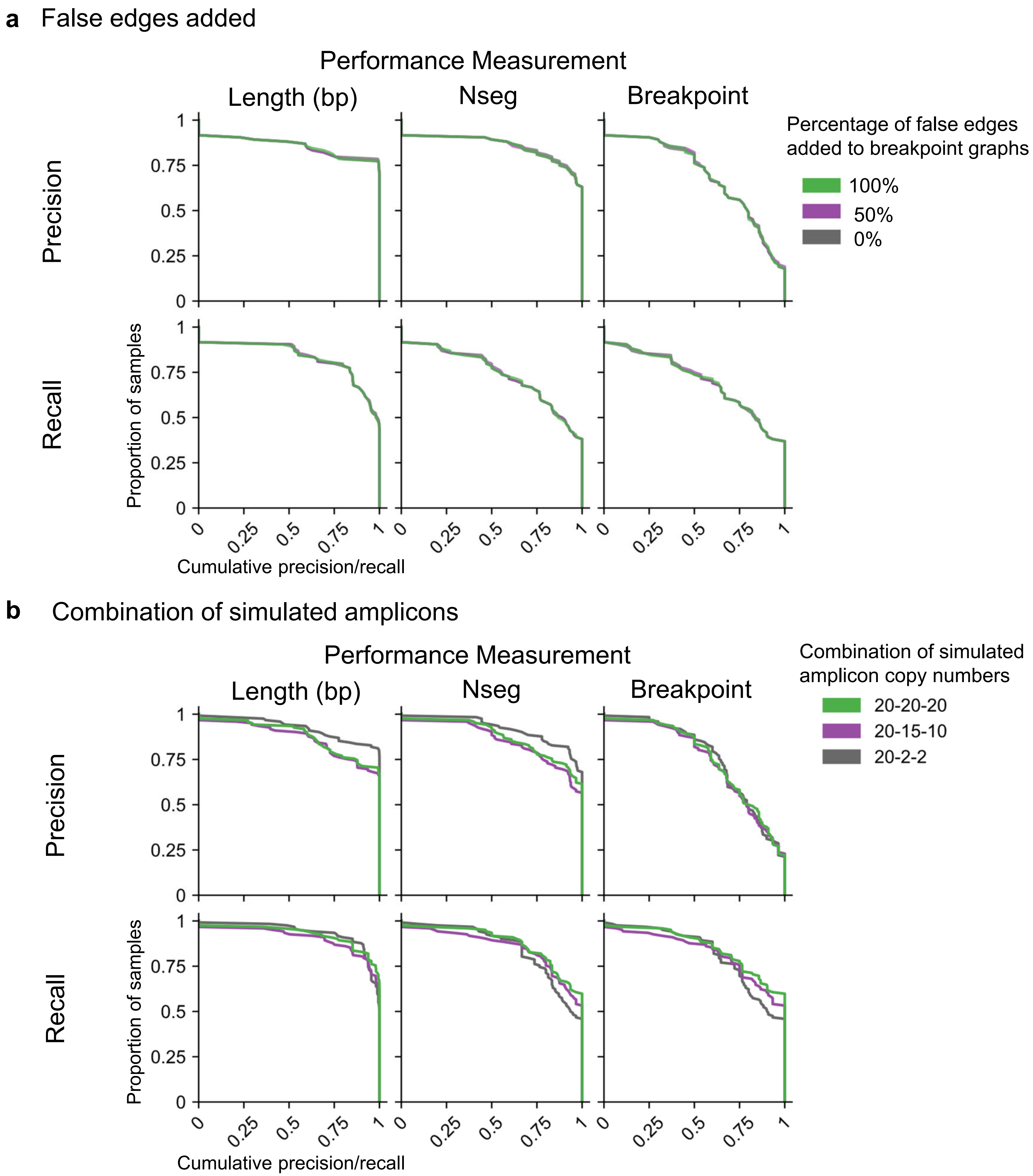

Supplementary Figure 5. AR performance on simulated data with false graph edges and amplicon mixtures.

a, Cumulative precision and recall curves measuring the performance of AR, using SegAligner for OM alignment, on simulated OM data with added false positive edges into simulated amplicon breakpoint graphs. OM assembly failures were not filtered from this panel. **b,** Cumulative precision and recall curves measuring the performance of AR using SegAligner for simulated heterogeneous mixtures of similar amplicons. OM assembly failures were not filtered from this panel. Source data are provided as a Source Data file.

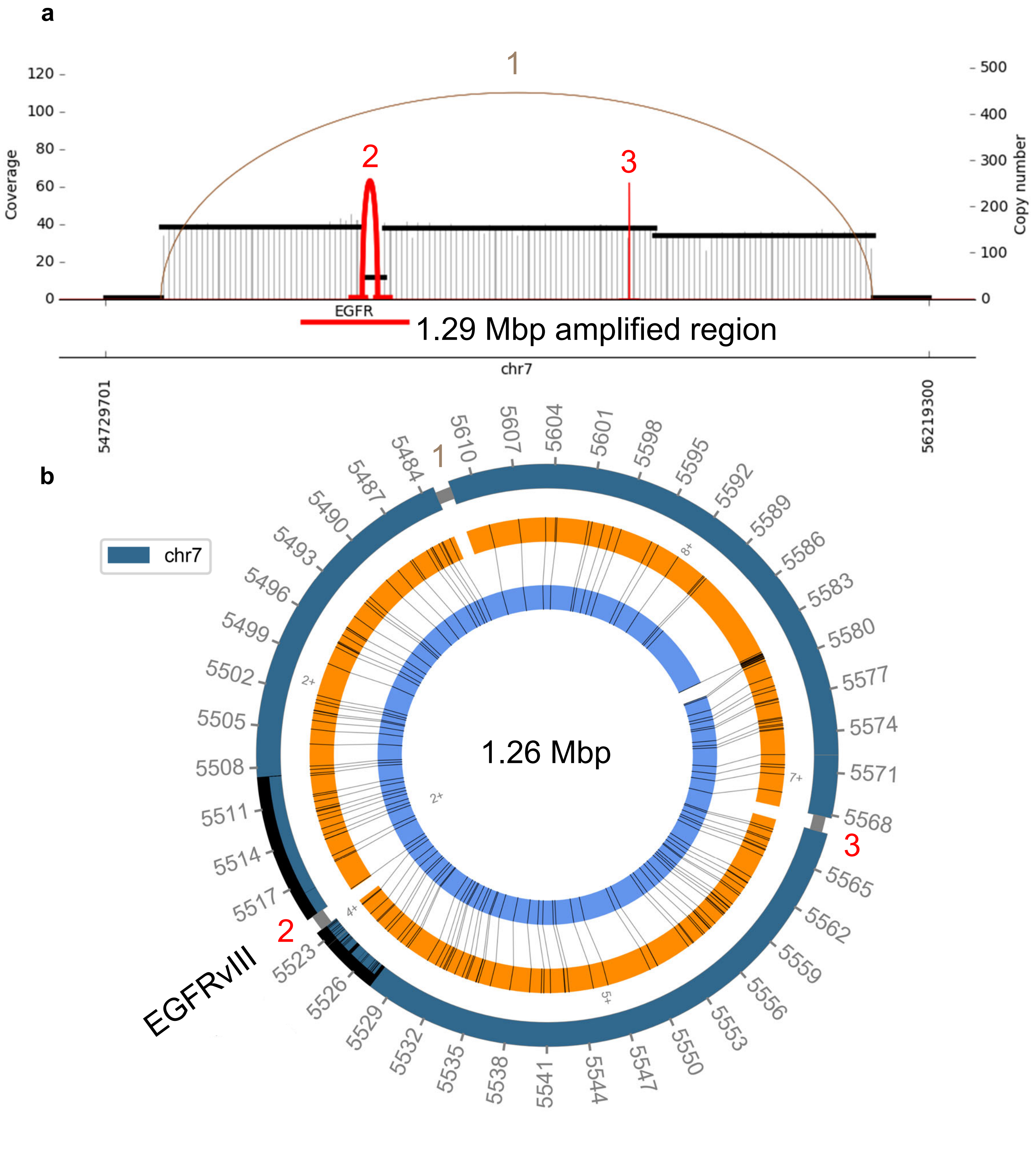

Supplementary Figure 6. AR reconstructs a circular ecDNA in GBM39.

a, AA detects a 1.29 Mbp amplified region of chr7 bearing *EGFR*, and indicates three breakpoint edges. Both *EGFR* and *EGFRvIII* appear to be amplified. **b**, AR (with SegAligner) reconstructs a circular 1.26 Mbp ecDNA in GBM39 using a single Bionano Irys contig. A legend for reading CycleViz AR figures can be found in Supplementary Figure 2a,b. Coordinate units on the labeled figure are scaled by 10 kbp. AR reconstructs a form of EGFR carrying the ~20 kbp vIII deletion, which is supported by the breakpoint graph.

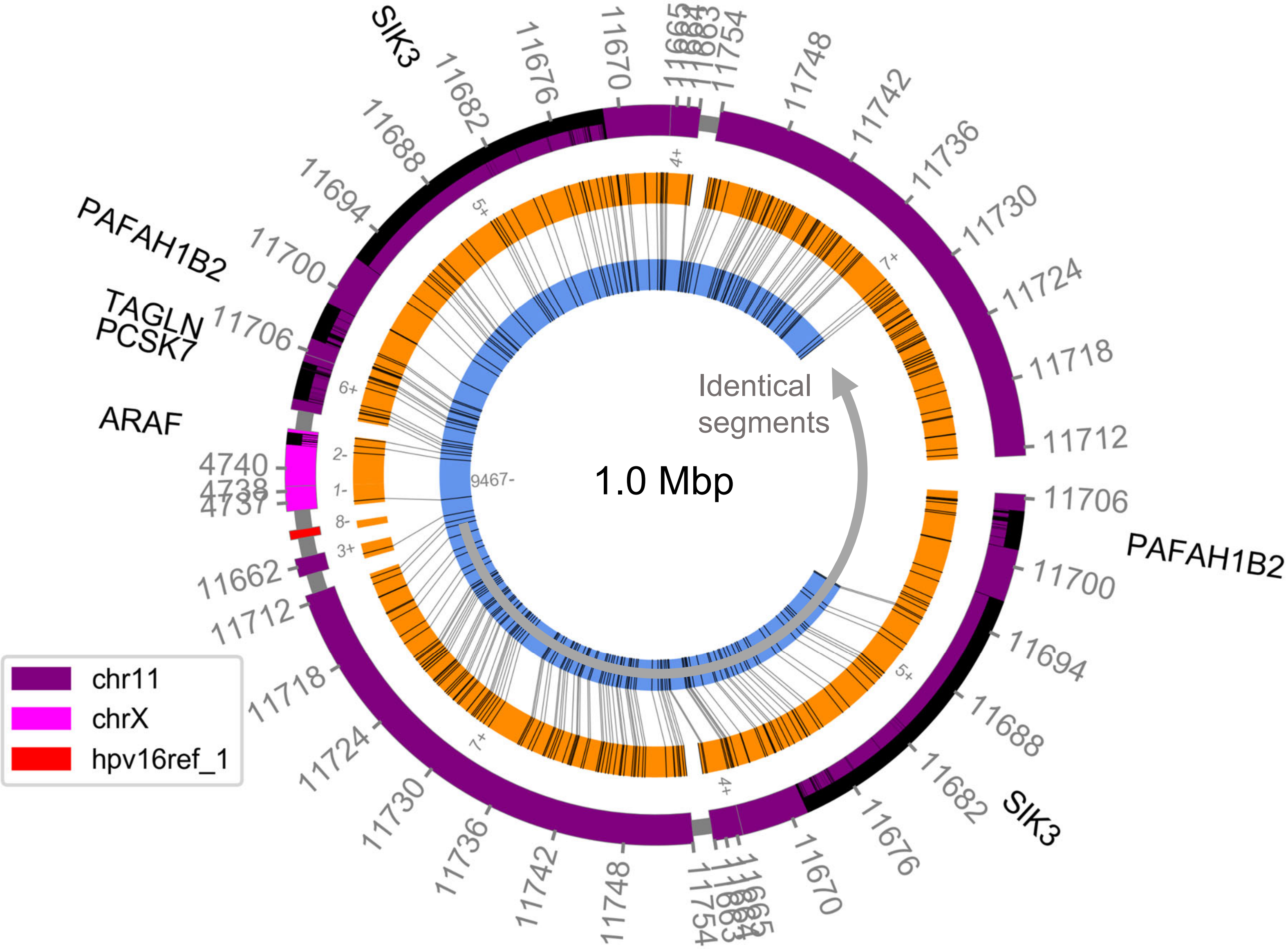

Supplementary Figure 7. AR reconstructs a simulated human-viral amplicon.

AR reconstruction of a simulated 1.0 Mbp circular ecDNA containing an integrated human papillomavirus-16 (HPV16). A legend for reading CycleViz AR figures can be found in Supplementary Figure 2a,b. Coordinate units on the labeled figure are scaled by 10 kbp. The grey arced arrow indicates identical overlap between segments assembled at the end of the structure which overlap to form the circular ecDNA structure.

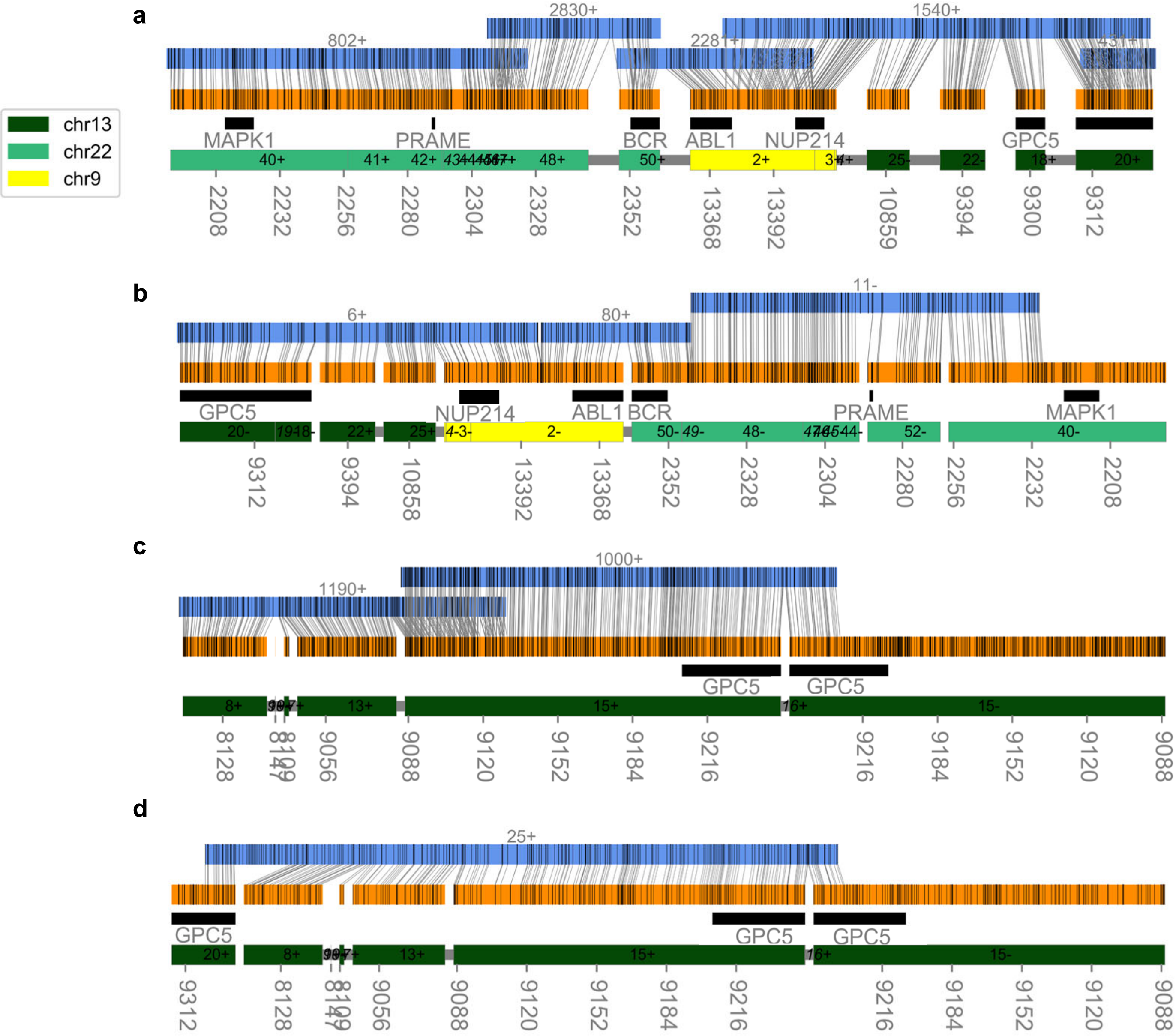

Supplementary Figure 8. AR reconstructions of K562 amplicon with Bionano Saphyr and Bionano Irys data.

a, AR reconstruction of *BCR-ABL1* amplicon in K562 using Bionano Saphyr data. A legend for reading CycleViz AR figures can be found in Supplementary Figure 2a,b. Coordinate units on the labeled figure are scaled by 10 kbp. **b,** AR reconstruction of BCR-ABL1 amplicon in K562 using Bionano Irys data. Amplicon was reconstructed in the reverse direction from panel **a** and includes a graph segment (19) which was not included in the Saphyr reconstruction. **c,** Additional AR reconstruction (using Bionano Saphyr data) from AA-detected amplified segments outside the reconstructions in panels **a** and **b**. **d**, Additional AR reconstruction (using Bionano Irys data) from AA-detected amplified segments outside the reconstructions in **a** and **b**, with the exception of the leftmost segment, 20, which appears at the leftmost end of the Irys reconstruction in panel **b.**

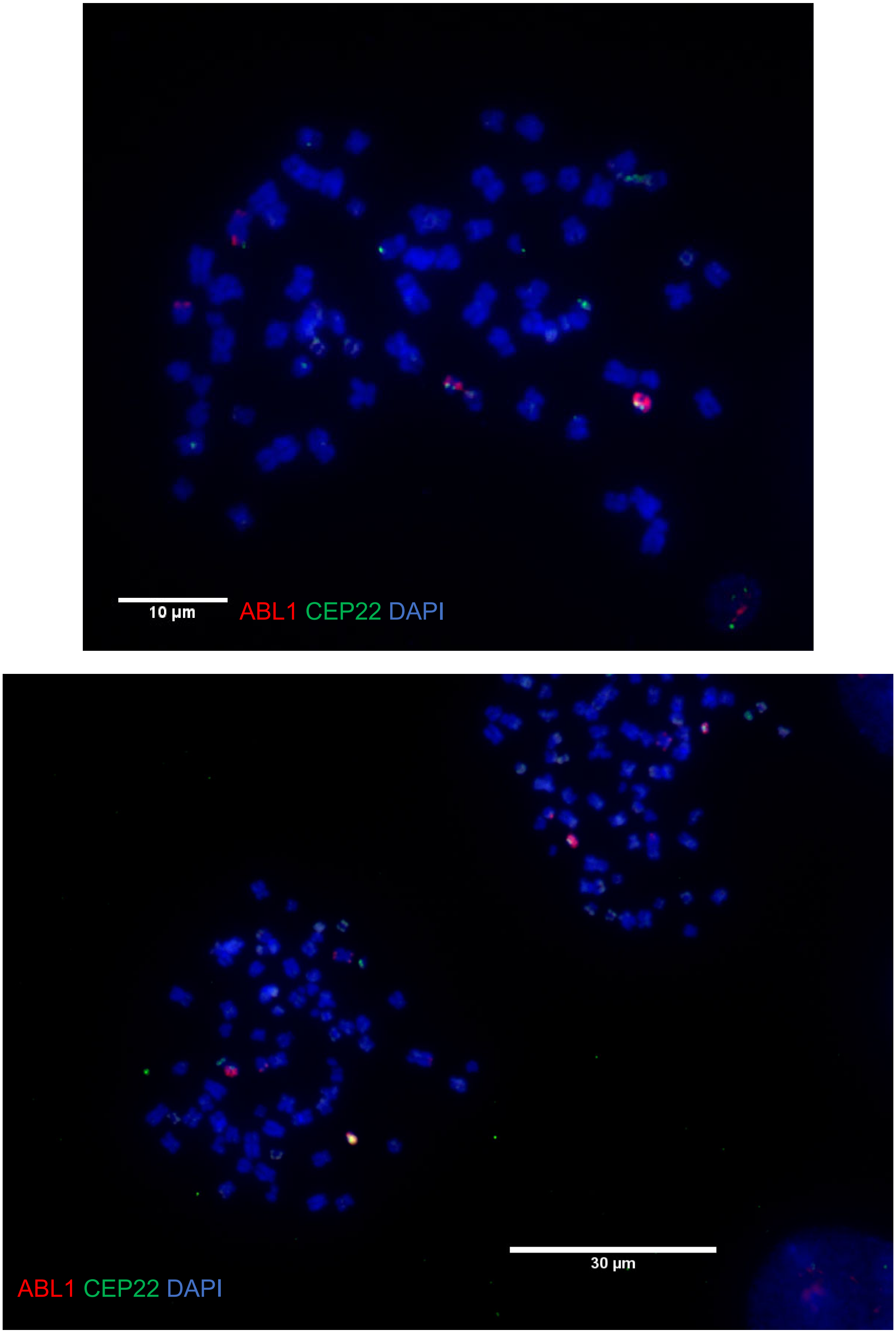

Supplementary Figure 9. Multi-FISH for K562.

Multi-FISH using confocal microscopy on DAPI-stained metaphase chromosomes in K562 cells. Centromeric repeat probe for chr22 (CEP22) and *ABL1* are stained in green and red, respectively. Two separate plate regions are shown.

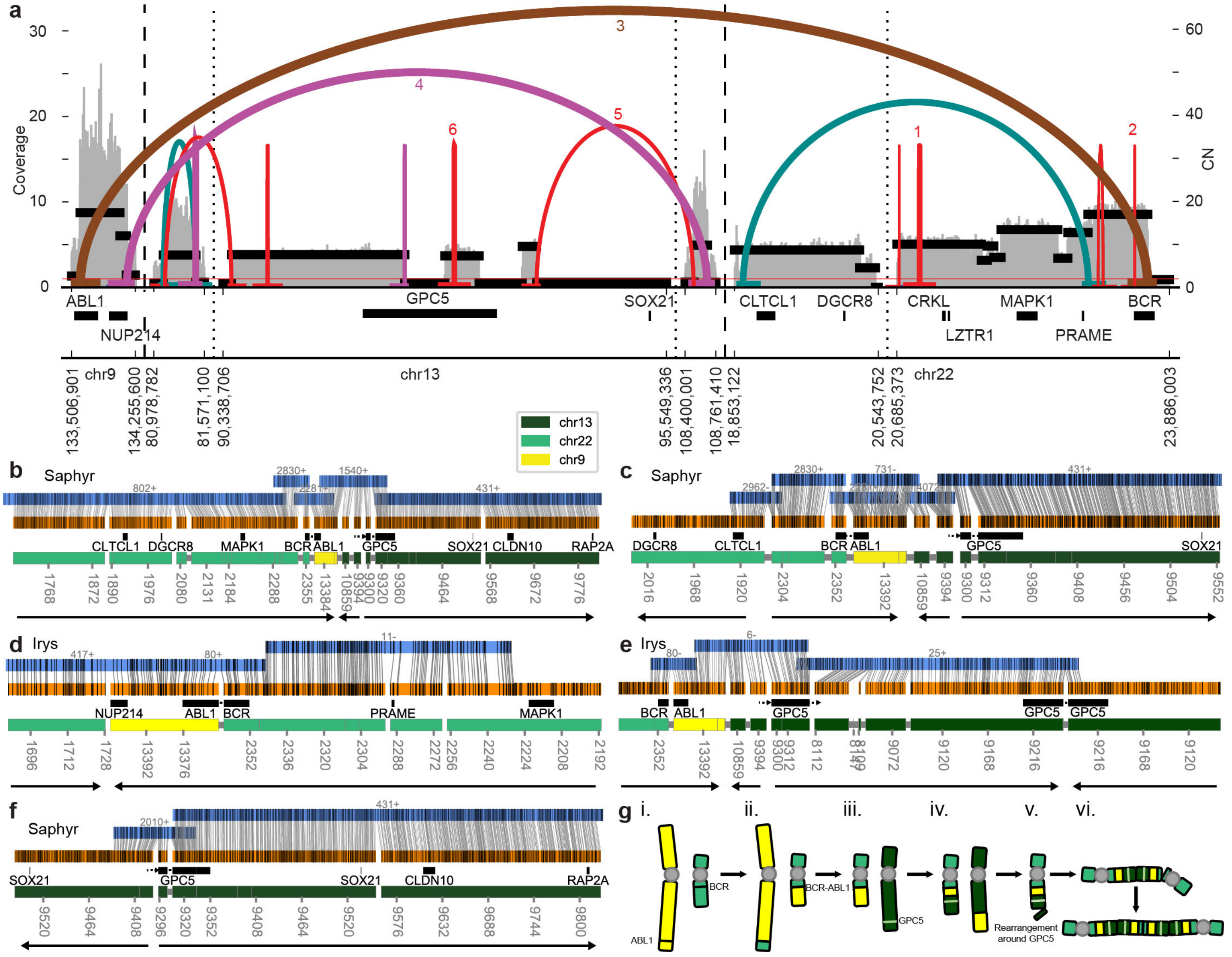

Supplementary Figure 10. Annotated AR reconstructions of K562 amplicon.

a, Reproduction of Fig. 3a with *SOX21* additionally labeled. **b**, Extended AR reconstruction of K562 *BCR-ABL1* amplicon using Saphyr data. **c**, AR reconstruction with Saphyr data supporting an alternate orientation for the genomic segment containing *CLTCL* as opposed to panel **b**. **d**, AR reconstruction with Irys data containing *BCR-ABL1* flanked by chr22 on both sides. **e**, AR reconstruction with Irys data containing *BCR-ABL1* with an inverted repeat of the 3' end of *GPC5*. **f**, AR reconstruction with Saphyr data shows inverted repeat of genomic segment containing *SOX21*. **g**, Hypothetical model for *BCR-ABL1* amplification in K562, involving the reciprocal translocation of q arms of chromosomes 9 and 22 to form the *BCR-ABL1* fusion (i-ii), then translocation of q arm of chromosome 13 with the *BCR-ABL1* chromosome (iii-iv) with rearrangement of the translocated regions from chr13 on the *BCR-ABL1* chromosome (v). Finally, inverted repeats observed in AR scaffolds can be explained by amplifications of *BCR-ABL1* through a dicentric chromosome model of amplification (vi).

a

Segment	A	B	C	D	E
Size (kbp)	1024	269	217	752	1176
Raw copy number	20.0	41.4	64.3	53.0	9.6
BFB normalized copy count	2	4	6	5	1

b

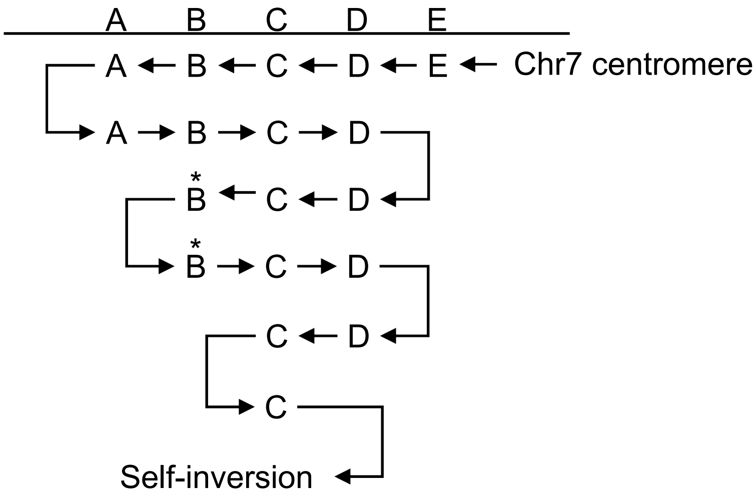

c

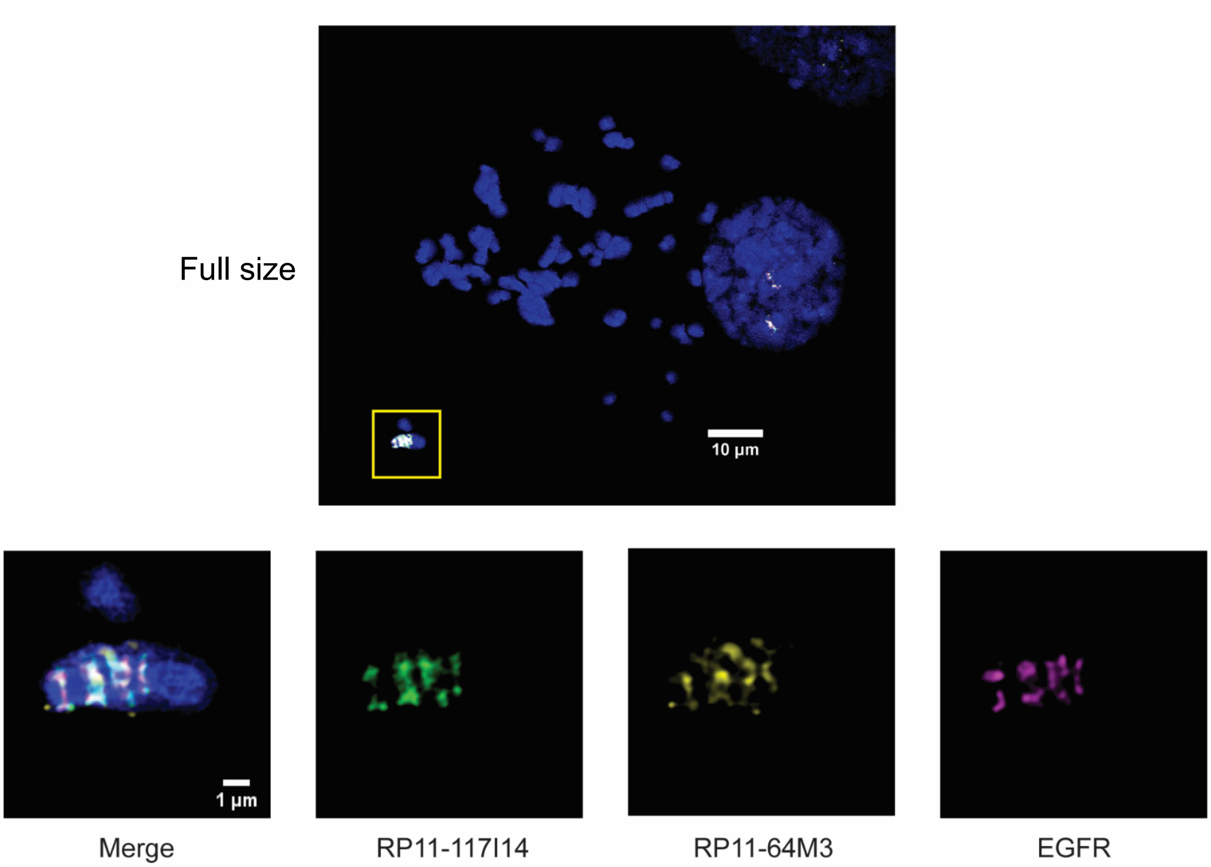

Supplementary Figure 11. BFB structure in HCC827.

a, Table of BFB amplicon segments showing size, raw copy number as estimated by AA, and normalized BFB count. **b**, A valid BFB string supported by both AA and AR, which contains interior structural variation (marked with an asterisk symbol, "*") **c**, Multi-FISH using super-resolution confocal microscopy on DAPI-stained metaphase chromosomes in HCC827. Brightness was decreased using ImageJ between full size and zoomed images. Probe RP11-64M3 corresponds to segment A, RP11-117I14 corresponds to segment C, and *EGFR* corresponds to segment D.

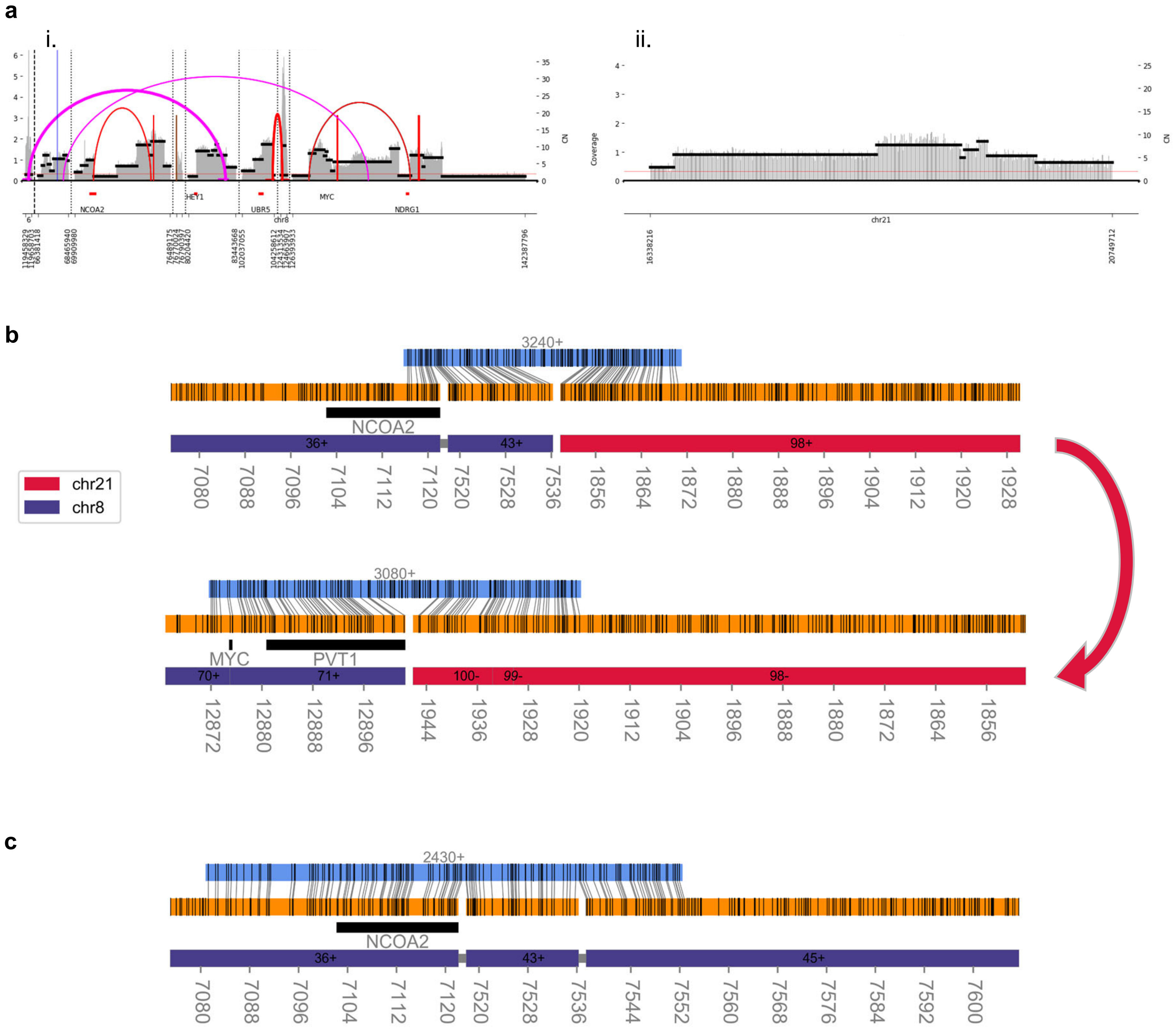

Supplementary Figure 12. Heterogeneous AR scaffolds identified in HCC827.

a, Two of five additional AA-generated breakpoint graphs for HCC827. The graph on the left (i) shows a complex structure of rearrangements, while the graph on the right (ii) shows distinct copy number changes without breakpoint graph edges. **b**, AR reconstruction of Bionano Saphyr scaffold joining segments from the graph labeled i in panel **a**, including *NCOA2* and *MYC*, to a segment of chr21 from the graph labeled ii in panel **a.** The scaffold on top in panel **b** can be joined to the scaffold on the bottom as the segment labeled 98 overlaps, as indicated with the curved red arrow. **c**, An alternate scaffold reconstructed by AR showing *NCOA2* joined to additional segments from chromosome 8.

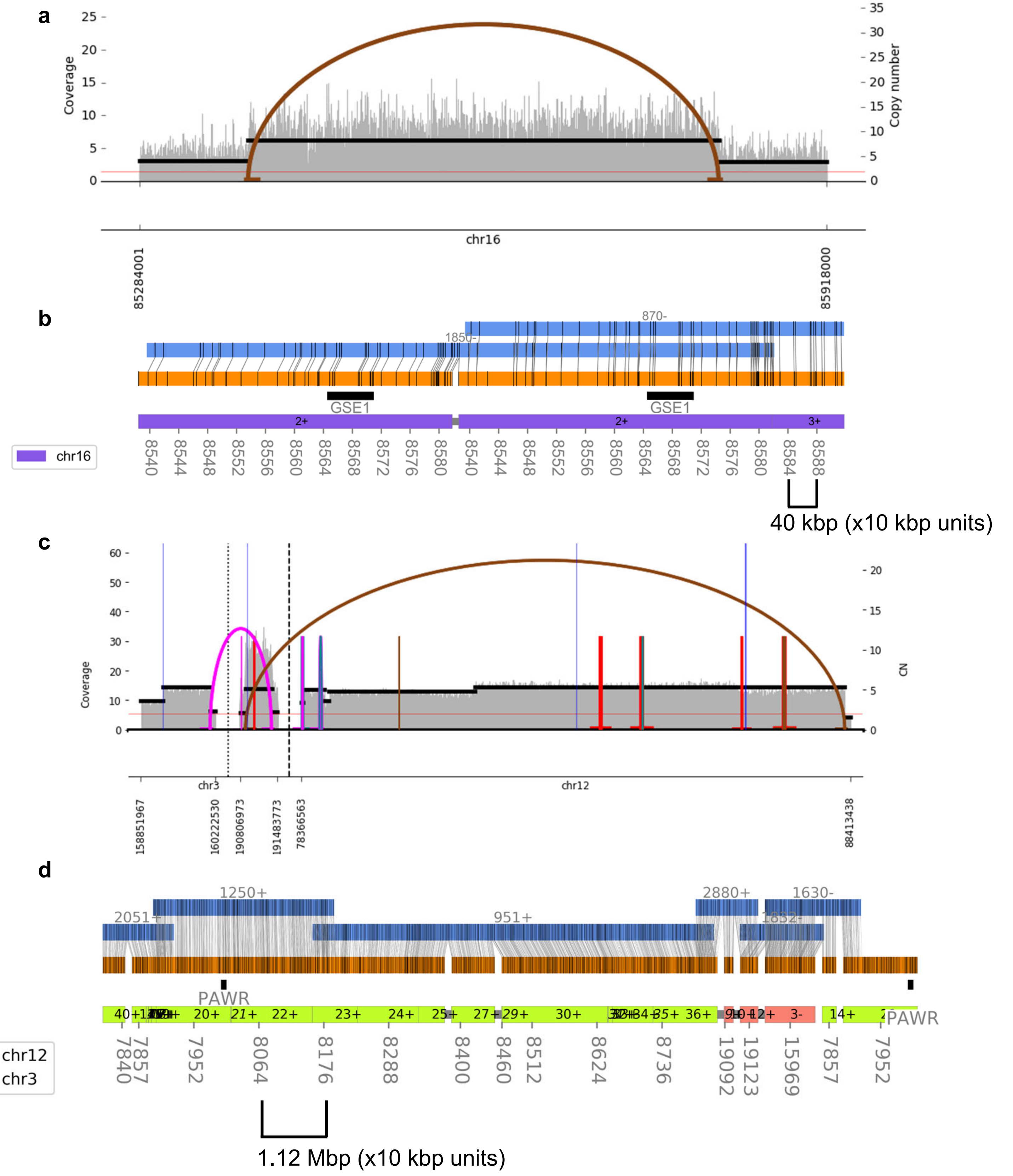

Supplementary Figure 13. AR reconstructions in T47D and CAKI-2.

a, In T47D, an AA-generated breakpoint graph visualization showing a 430 kbp amplified region with a single breakpoint edge connecting the ends of the amplicon. **b**, In T47D, AR reconstruction of the amplicon shows a 967 kbp region capturing the segmental tandem duplication involving *GSE1*. **c**, In CAKI-2, an AA-generated breakpoint graph visualization showing a complex amplified rearrangement joining segments (12.0 Mbp in total) from chr12 and chr3 in CAKI-2. **d**, In CAKI-2, AR-reconstructed 13.1 Mbp amplicon containing segments from chr12 and 3, including interior deletions and multiple copies of *PAWR*.

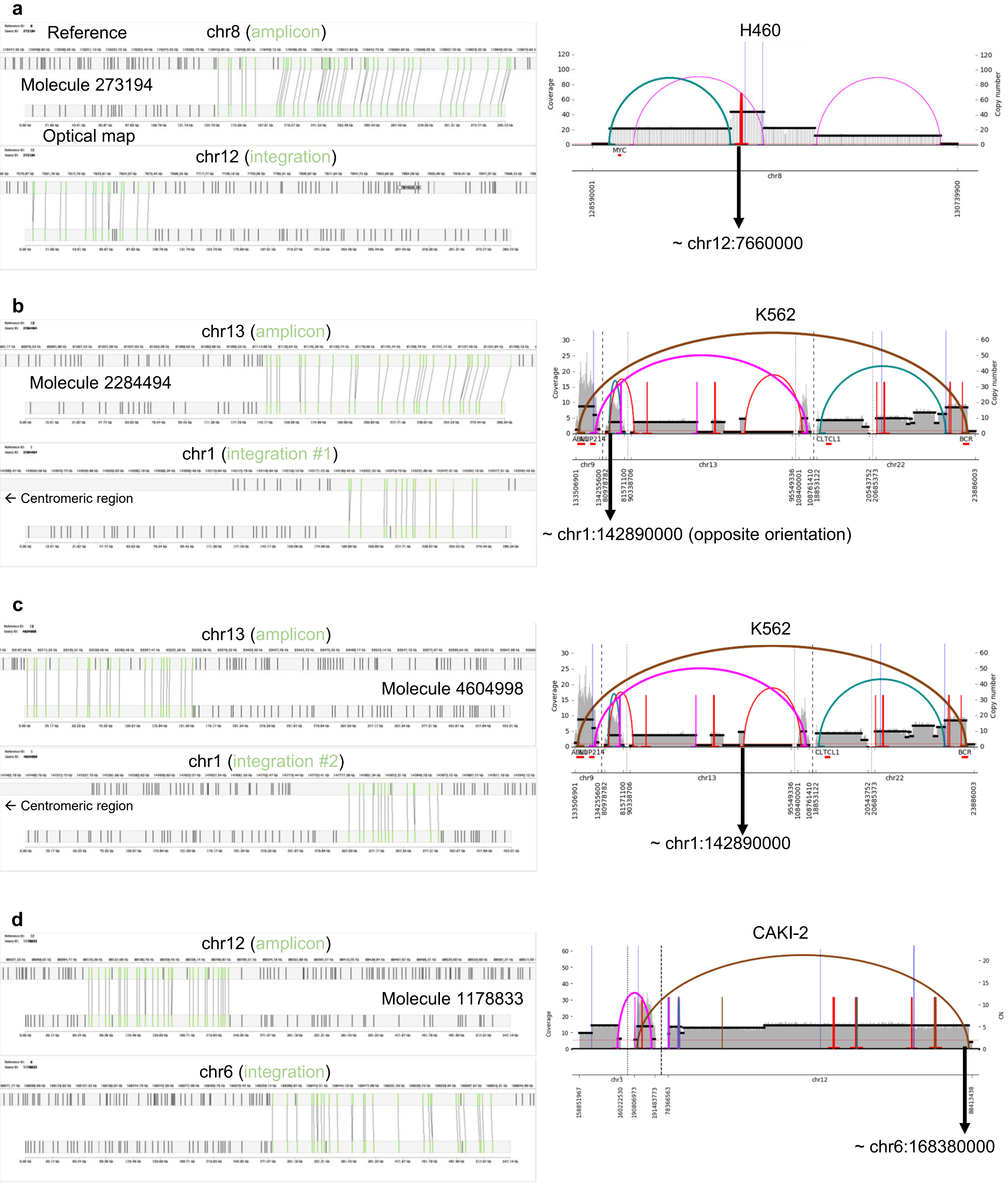
a
Reference
chr8 (amplicon)
Molecule 273194
Optical map
chr12 (integration)
H460
Coverage
Copy number
MYC
chr8
128590001
130739900
~ chr12:7660000
b
chr13 (amplicon)
Molecule 2284494
chr1 (integration #1)
← Centromeric region
K562
CLTCL1
BCR
chr9
chr13
chr22
~ chr1:142890000 (opposite orientation)
c
chr13 (amplicon)
Molecule 4604998
chr1 (integration #2)
← Centromeric region
K562
~ chr1:142890000
d
chr12 (amplicon)
Molecule 1178833
chr6 (integration)
CAKI-2
chr3
chr12
~ chr6:168380000

Supplementary Figure 14. Integration points of focal amplifications.

a, (Left) An example of a supporting alignment between an amplified region of chr8 (top) and a non-amplicon region on chr12 (bottom) in H460. (Right) The approximate integration point on the amplicon is indicated with a black arrow pointing at the approximate integration location. **b**, (Left) An example of a supporting alignment between an amplified region of chr13 (top) and a non-amplicon region on chr1 in K562 (bottom). An arrow pointing towards the centromere is also present due to the proximity of the integration point to the centromeric region (~ 80kbp). (Right) The approximate integration point on the amplicon is indicated with a black arrow pointing at the approximate integration location. **c**, (Left) An example of a supporting alignment between an amplified region of chr13 (top) and a non-amplicon region on chr1 in K562 (bottom). An arrow pointing towards the centromere is also present due to the proximity of the integration point to the centromeric region (~ 240kbp). (Right) The approximate integration point on the amplicon is indicated with a black arrow pointing at the approximate integration location. **d**, (Left) An example of a supporting alignment between an amplified region of chr12 (top) and a non-amplicon region on chr6 (bottom) in CAKI-2. (Right) The approximate integration point on the amplicon is indicated with a black arrow pointing at the approximate integration location.

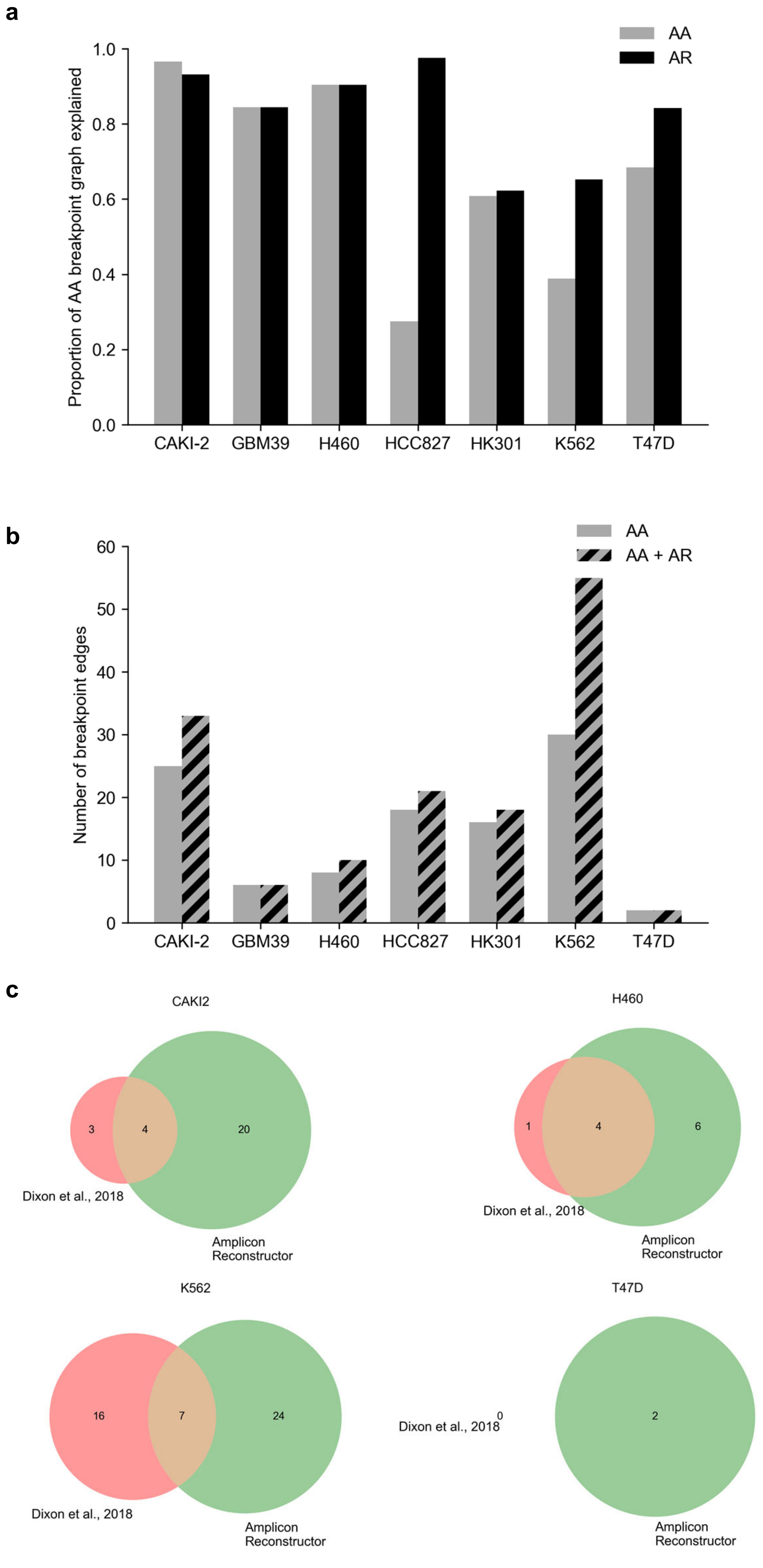
a
AA
AR
Proportion of AA breakpoint graph explained
1.0
0.8
0.6
0.4
0.2
0.0
CAKI-2
GBM39
H460
HCC827
HK301
K562
T47D
b
AA
AA + AR
Number of breakpoint edges
60
50
40
30
20
10
0
CAKI-2
GBM39
H460
HCC827
HK301
K562
T47D
c
CAKI2
3
4
20
Dixon et al., 2018
Amplicon Reconstructor
H460
1
4
6
Dixon et al., 2018
Amplicon Reconstructor
K562
16
7
24
Dixon et al., 2018
Amplicon Reconstructor
T47D
0
Dixon et al., 2018
2
Amplicon Reconstructor

Supplementary Figure 15. Comparing AR's results with AA output and Dixon et al. results

a, Comparison between the heaviest path/cycle generated by AA (grey) and the heaviest path/cycle generated by AR showing the proportion of amplified content in the breakpoint graph which is explained in the path/cycle for all samples studied by AA and AR. **b**, The number of breakpoint graph edges suggested by AA (grey) and the total number of edges inferred by AA and AR (black and grey stripes) as measured by the union of the junctions inferred by AR and the edges in the AA graph. Source data for **a** and **b** are provided as a Source Data file. **c**, Venn diagrams of the overlap between breakpoints detected by the Dixon et al. study (high confidence integrated breakpoints) and the breakpoints detected by AR for the amplicon regions analyzed by AR for CAKI-2, H460, K562 and T47D.

Supplementary References

1. Dixon, J. R. *et al.* Integrative detection and analysis of structural variation in cancer genomes. *Nat. Genet.* **50**, 1388–1398 (2018).
