# Supplementary material for: AmpliconReconstructor integrates NGS and optical mapping to resolve the complex structures of focal amplifications
Source: Nat Commun. 2020 Sep 1;11:4374. doi: 10.1038/s41467-020-18099-z (PMC7463033; doi:10.1038/s41467-020-18099-z)
Supplement: Supplementary file 3 — Description of Additional Supplementary Files [file 41467_2020_18099_MOESM3_ESM.pdf]

Description of Additional Supplementary Files

File Name: Supplementary Data 1
Description: SRA accession numbers used for WGS data, OM molecule length statistics and OM assembly statistics for cell lines in this study.

File Name: Supplementary Data 2
Description: AR performance on simulated data. Includes comparison of SegAligner and other OM alignment methods, amplicon mixture performance, false graph edge performance, profile of simulated amplicons, and performance on *de novo* simulated amplicons.

File Name: Supplementary Data 3
Description: Focal amplification breakpoints identified in both AR and AA for cell lines in this study and estimated integration point locations for focal amplifications.

File Name: Supplementary Data 4
Description: AR amplicon reconstruction paths and summary statistics about AR reconstructions for cell lines in this study.

File Name: Supplementary Data 5
Description: Default parameters for AR and SegAligner.
