# Supplementary material for: AmpliconReconstructor integrates NGS and optical mapping to resolve the complex structures of focal amplifications
Source: Nat Commun. 2020 Sep 1;11:4374. doi: 10.1038/s41467-020-18099-z (PMC7463033; doi:10.1038/s41467-020-18099-z)
Supplement: Supplementary file 9 — Reporting Summary [file 41467_2020_18099_MOESM9_ESM.pdf]

nature research

Corresponding author(s): Vineet Bafna
Jens Luebeck

Last updated by author(s): Jul 14, 2020

Reporting Summary

Nature Research wishes to improve the reproducibility of the work that we publish. This form provides structure for consistency and transparency in reporting. For further information on Nature Research policies, see our Editorial Policies and the Editorial Policy Checklist.

Statistics

For all statistical analyses, confirm that the following items are present in the figure legend, table legend, main text, or Methods section.

n/a	Confirmed	
☐	☒	The exact sample size (*n*) for each experimental group/condition, given as a discrete number and unit of measurement
☐	☒	A statement on whether measurements were taken from distinct samples or whether the same sample was measured repeatedly
☐	☒	The statistical test(s) used AND whether they are one- or two-sided<br>*Only common tests should be described solely by name; describe more complex techniques in the Methods section.*
☒	☐	A description of all covariates tested
☒	☐	A description of any assumptions or corrections, such as tests of normality and adjustment for multiple comparisons
☒	☐	A full description of the statistical parameters including central tendency (e.g. means) or other basic estimates (e.g. regression coefficient) AND variation (e.g. standard deviation) or associated estimates of uncertainty (e.g. confidence intervals)
☐	☒	For null hypothesis testing, the test statistic (e.g. *F*, *t*, *r*) with confidence intervals, effect sizes, degrees of freedom and *P* value noted<br>*Give P values as exact values whenever suitable.*
☒	☐	For Bayesian analysis, information on the choice of priors and Markov chain Monte Carlo settings
☒	☐	For hierarchical and complex designs, identification of the appropriate level for tests and full reporting of outcomes
☒	☐	Estimates of effect sizes (e.g. Cohen's *d*, Pearson's *r*), indicating how they were calculated

Our web collection on statistics for biologists contains articles on many of the points above.

Software and code

Policy information about availability of computer code

Data collection	Bionano AutoDetect 2.1.4<br>Bionano IrysSolve 2.1<br>Bionano Access 1.2.1

Data analysis	Bionano RefAligner (v5122)<br>Bionano Assembler (v5122)<br>SCNVSim (1.3.1)<br>BWA-MEM (0.7.17-r1188)<br>SAMtools (0.1.19-96b5f2294a)<br>Freebayes (v1.3.1-17-gaa2ace8)<br>Canvas CNV caller (1.39.0.1598)<br>CytoscapeJS (3.2.14)<br>Scikit: https://scikit-learn.org/stable/ (1.0)<br>MapOptics: https://github.com/FadyMohareb/mapoptics (1.0)<br>ReadDepth: https://github.com/chrisamiller/readDepth (1.0)<br>AmpliconArchitect: https://github.com/virajbdeshpande/AmpliconArchitect (1.0)<br>PrepareAA: https://github.com/jluebeck/PrepareAA (1.0)<br>AmpliconReconstructor (& SegAligner): https://github.com/jluebeck/AmpliconReconstructor (1.0)<br>CycleViz: https://github.com/jluebeck/CycleViz (1.0)<br>ScaffoldGraphViewer: https://github.com/jluebeck/ScaffoldGraphViewer (1.0)<br>ImageJ (1.52a)

For manuscripts utilizing custom algorithms or software that are central to the research but not yet described in published literature, software must be made available to editors and reviewers. We strongly encourage code deposition in a community repository (e.g. GitHub). See the Nature Research guidelines for submitting code & software for further information.

Data

Policy information about availability of data

All manuscripts must include a data availability statement. This statement should provide the following information, where applicable:
- Accession codes, unique identifiers, or web links for publicly available datasets
- A list of figures that have associated raw data
- A description of any restrictions on data availability

The AA-generated breakpoint graphs for the cell lines in this study are available on figshare with the identifier doi:10.6084/m9.figshare.11691798 [https://figshare.com/articles/AA_breakpoint_graphs/11691798]. The FISH data that support the findings of this study are available on figshare with the identifier doi:10.6084/m9.figshare.11691774 [https://figshare.com/articles/FISH/11691774]. Assembled Bionano contigs that support the findings of this study have been deposited in GenBank with Bioproject codes PRJNA602907 [https://www.ncbi.nlm.nih.gov/bioproject/PRJNA602907/] and PRJNA506071 [https://www.ncbi.nlm.nih.gov/bioproject/PRJNA506071]. The source data underlying Figure 1i, and Supplementary Figures 1d, 4, 5a & b, 15a & b are provided as a Source Data file. The remaining data are available in the Article, Supplementary Information or available from the authors upon reasonable request.

Field-specific reporting

Please select the one below that is the best fit for your research. If you are not sure, read the appropriate sections before making your selection.

☒ Life sciences ☐ Behavioural & social sciences ☐ Ecological, evolutionary & environmental sciences

For a reference copy of the document with all sections, see nature.com/documents/nr-reporting-summary-flat.pdf

Life sciences study design

All studies must disclose on these points even when the disclosure is negative.

Sample size	We studied seven cancer cell lines in this study. These cell lines represent a diverse set of cancer cell lines carrying focal amplifications with different generative signatures. The sample size for this study was determined by the availability of Bionano datasets. The sample size of the simulations - which are used to show the accuracy of our method - is 85 samples, and this number of cases was selected as it was the largest number of putative amplicon structures available to us. These sample sizes are sufficient as they encompass a large variety of different amplicon cases and the variance of AR's performance across the cases was observed to be small in comparison to the sample size.
Data exclusions	Cell line LNCAP was excluded as we did not detect any focal amplifications to analyze. Cell line HK359 was excluded due to insufficient OM data.
Replication	All FISH experiments involved the analysis of at least three independent images and representative results are shown in the figures present in the study. All attempts at replication in our study were successful. Our tool creates deterministic output and thus its output is also replicable. We further detail the successful replication of the computational results below, even when using slightly different approaches for generating the input for the tool.<br><br>We generated breakpoint graphs using seeding from two different CNV callers ReadDepth and Canvas, and achieved remarkably similar results. Furthermore, we tried running AmpliconReconstructor on the same breakpoint graph, OM data sets, and parameters multiple times, and achieved identical outputs, indicating that the results produced by this method are deterministic. For one of our samples, K562, we generated OM data using two different Bionano OM instruments, Saphyr and Irys, and achieved highly similar results. We expect that a third party running our tool on the data present here would produce the same output as we show in this paper.
Randomization	We developed a computational technique for reconstruction of focal amplifications. Samples were not grouped in our study design and thus

	randomization into groups is not applicable to this study design.
Blinding	We developed a computational technique for reconstruction of focal amplifications, and thus our study design did not warrant the use of any blinding.

Reporting for specific materials, systems and methods

We require information from authors about some types of materials, experimental systems and methods used in many studies. Here, indicate whether each material, system or method listed is relevant to your study. If you are not sure if a list item applies to your research, read the appropriate section before selecting a response.

Materials & experimental systems

n/a	Involved in the study
☒	☐ Antibodies
☐	☒ Eukaryotic cell lines
☒	☐ Palaeontology and archaeology
☒	☐ Animals and other organisms
☒	☐ Human research participants
☒	☐ Clinical data
☒	☐ Dual use research of concern

Methods

n/a	Involved in the study
☒	☐ ChIP-seq
☒	☐ Flow cytometry
☒	☐ MRI-based neuroimaging

Eukaryotic cell lines

Policy information about cell lines

Cell line source(s)	We did not create any new cell lines for this study. K562, NCI-H460 and HCC827 were provided by Andrew Shiau. The original source of K562 and NCI-H460 was the NCI-60 cancer cell line panel and HCC827 was previously from ATCC. CAKI-2 and T47D were provided by Bionano Genomics. HK301 was provided by Harvey Kornblum, who was the original source. GBM39 was provided by David James, who was the original source.
Authentication	Cell lines were not authenticated.
Mycoplasma contamination	Cell lines were not tested.
Commonly misidentified lines (See ICLAC register)	No commonly misidentified cell lines were used in the study.
